# Rutin inhibits DRP1-mediated mitochondrial fission and prevents ethanol-induced hepatotoxicity in HepG2 cells and zebrafish

**DOI:** 10.1080/19768354.2021.1882565

**Published:** 2021-02-11

**Authors:** Youngsook Choi, Heymin Seo, Mina Cho, Joohee Kim, Hak Suk Chung, Icksoo Lee, Min Jung Kim

**Affiliations:** a Research Institute of Women’s Health, Sookmyung Women’s University, Seoul, Korea; b Department of Biological Sciences, Sookmyung Women’s University, Seoul, Korea; c Center for Teragnosis, Biomedical Research Institute, Korea Institute of Science and Technology, Seoul, Korea; d Division of Bio-Medical Science & Technology, KIST School, Korea University of Science and Technology, Seoul, Korea; e College of Medicine, Dankook University, Cheonan, Korea

**Keywords:** Rutin, ethanol, DRP1, zebrafish, mitochondria

## Abstract

Excessive alcohol consumption causes the cellular and tissue damage. The toxic metabolites of ethanol are harmful to multiple organ systems, such as the central nervous system, skeletal muscles, and liver, and cause alcohol-induced diseases like cancer, as well as induce hepatotoxicity, and alcoholic myopathy. Alcohol exposure leads to a surge in hepatic alcohol metabolism and oxygen consumption, a decrease in hepatic ATP, and the rapid accumulation of lipid within hepatocytes. Several pathologies are closely linked to defective mitochondrial dynamics triggered by abnormal mitochondrial function and cellular homeostasis, raising the possibility that novel drugs targeting mitochondrial dynamics may have therapeutic potential in restoring cellular homeostasis in ethanol-induced hepatotoxicity. Rutin is a phytochemical polyphenol known to protect cells from cytotoxic chemicals. We investigated the effects of rutin on mitochondrial dynamics induced by ethanol. We found that rutin enhances mitochondrial dynamics by suppressing mitochondrial fission and restoring the balance of the mitochondrial dynamics. Mitochondrial elongation following rutin treatment of ethanol exposed cells was accompanied by reduced DRP1 expression. These data suggest that rutin plays an important role in remodeling of mitochondrial dynamics to alleviate hepatic steatosis and enhance mitochondrial function and cell viability.

## 1. Introduction

Alcohol consumption is a high risk factor associated with more than 200 diseases, such as alcohol fatty liver diseases (AFLD), chronic kidney disease (CKD), cardiovascular disorders, respiratory diseases, and cancers (Shield and Rehm 2019; Shin 2020). AFLD is a major cause of mortality worldwide and heavy ethanol consumption embraces a wide spectrum of AFLD ranging from mild steatosis, hepatitis, fibrosis/cirrhosis to hepatocellular carcinoma (Shield and Rehm 2019). Even though the most effective intervention against AFLD is complete abstinence from alcohol, the development of targeted therapies is imperative for severe AFLD and patients who cannot abstain from alcohol. The pathobiology of AFLD is not fully understood and the limitations of existing animal models of AFLD hinder the development of targeted therapies.

Mitochondria are essential cellular organelles. Mitochondrial dysfunction and oxidative stress are associated with NAFLD and AFLD (Nassir and Ibdah 2014b; Shin et al. 2020). Mitochondrial dynamics, biogenesis, and mitophagy regulate mitochondrial morphology, quality, and energy homeostasis during alcohol-induced cytotoxicity (Mansouri et al. 2018). Inhibition of β-oxidation by ethanol interferes with the regulation of glucose/lipid metabolism and induces endoplasmic reticulum stress, which increases cholesterol synthesis and trafficking into mitochondria, leading to steatosis (Nassir and Ibdah 2014a). Mitochondria contain dynamic networks that are regulated by fusion and fission processes. Fused mitochondria provide higher ATP levels for cellular homeostasis and fragmented mitochondria reduce mitochondrial respiration (Dai and Jiang 2019). Inhibition of mitochondrial fission suppresses hepatic oxidative stress as well as steatosis in nonalcoholic fatty liver disease (NAFLD) (Galloway et al. 2014). Mitochondrial fusion is primarily controlled by three GTPases, including mitofusin 1 (MFN1), mitofusin 2 (MFN2), and optic atrophy 1 (OPA1), while mitochondrial fission is primarily regulated by a single GTPase dynamin-related protein 1 (DRP1) (Dai and Jiang 2019). Dysregulated activities of these GTPases disrupt mitochondrial dynamics and cellular metabolism connected with mitochondria related pathologies such as NARP (Neuropathy, Ataxia, and Retinitis Pigmentosa), MILS (Maternally Inherited Leigh Syndrome), CMT2A (Charcot-Marie-Tooth disease type 2A), and neurodegenerative diseases (Dai and Jiang 2019). While several proteins have been suggested as DRP1 inhibitors, their exact mechanisms of action and mitochondria-related pathology are not fully understood**.**

Rutin is a phytochemical with anti-oxidative, anti-inflammatory, and anti-cancer activities and is known to protect and even rescue cells from the toxicity induced by ethanol, methylmercury, radiation, and hydrogen peroxide. Recent studies have been focused on the hepato-protective and neuro-protective roles of rutin (Ganeshpurkar and Saluja 2017; Enogieru et al. 2018). It suppresses apoptosis by improving mitochondrial respiration, mitochondrial membrane potential, and mitochondrial biogenesis (Enogieru et al. 2018). While the hepato- and neuro-protective properties of rutin were reported, the mechanisms of rutin in mitochondrial dynamics remain unknown. Since mitochondrial fusion and fission is strongly correlated with mitochondrial function and lipid metabolism, rutin may improve mitochondrial function by regulating mitochondrial dynamics.

The aim of our study was to investigate the effect of the flavonoid rutin on lipid droplet accumulation, steatosis, and dysregulation of mitochondrial dynamics under ethanol exposure in HepG2 and live zebrafish larvae.

## 2. Materials and methods

### 2.1. General reagents and cell culture

Rutin (Cat. No. R5143), ethanol (Cat. No. 02870), and other chemicals were purchased from Merck unless otherwise specified. HepG2 cells were cultured in MEM media with 10% fetal bovine serum, 100 U/mL penicillin and 100 μg/mL streptomycin and incubated at 37°C containing 5% CO_2_ in air.

### 2.2. Zebrafish care and maintenance

All zebrafish husbandry and experimental protocols complied with institutional guidelines and were approved by local ethics boards (Sookmyung Women’s University Animal Care and Use Committee, SMWU-IACUC-1712-036). Zebrafish were maintained under standard conditions at 28.5°C with a 14 h light/10 h dark cycle. Embryos were obtained from natural crosses between *TG(mito:EGFP)* zebrafish (Kim et al. 2008).

### 2.3. Cell viability assay

Cells were treated with ethanol and/or rutin for 24 h. Cell viability was determined with 3-(4,5-dimethylthiazol-2-yl)-2,5-diphenyltetrazolium bromide (MTT; AMRESCO) (Ly et al. 2020). The absorbance was measured at 550 nm with a SpectraMax® i3x microplate reader (VWR).

### 2.4. Oil Red O staining of HepG2 cells and zebrafish larvae

HepG2 cells cultured onto 0.1% poly-D-lysine-coated coverslips were stained with 0.3% Oil Red O solution. Zebrafish larvae were fixed with 4% PFA, bleached with 1% KOH/1.5% H_2_O_2_ solution, and stained with 0.3% Oil Red O solution. Images of HepG2 cells were acquired with an inverted phase-contrast microscope (Leica microsystems, Leitz DMRB) and those of zebrafish larvae using a bright-field dissecting microscope (Nikon SMZ1500). Widths (or areas) of stained lipid were analyzed using ImageJ.

### 2.5. Confocal live imaging and measurement of mitochondrial length in HepG2 cells and zebrafish larvae

Live images of red fluorescence protein DsRed2-MLS vector transfected HepG2 cells using the GENE-Fect^TM^ transfection reagent (Translab) were obtained with an LSM 700 confocal laser microscope (Carl Zeiss) and presented as a stacked image (10 images; interval thickness: 1 μm). Z-series of images (10 images; interval thickness: 1 μm) of *TG(mito:EGFP)* zebrafish larvae were collected with an LSM 700 confocal laser microscope and presented as a stacked image. The average length of 10 mitochondria was determined from 50 individual cells in 3 independent experiments.

### 2.6. RT-PCR

Total RNA was reverse transcribed with a GoScript reverse transcription kit (Cat. No. A5000, Promega). The qPCR was performed in a ABI7500 real-time PCR system (Merck) with EvaGreen qPCR Supermix (Solis BioDyne). Expression of *opa1*, *drp1, mfn*2, *c/ebpα* and *pparγ* was normalized to that of the corresponding *gapdh*. Primer sequences are listed in Supplementary Table 1.

### 2.7. Western blot

Whole-cell lysates (30 μg) were loaded on 10–15% SDS-PAGE, transferred electrophoretically to a PVDF membrane, incubated with antibodies against GAPDH (Merck), DRP1 (Santa Cruz), MFN2 (Santa Cruz), and then incubated with the HRP-conjugated secondary antibody (Merck) (Ho et al. 2019; Long et al. 2019). Signals were detected with SuperSignal West Pico Chemiluminescent Substrate (Merck) using the Odyssey imaging system (LI-COR).

### 2.8. Statistical analysis

The data are presented as the mean and standard deviation of the results from three independent experiments (*n* = 3). The statistical significance of the experimental differences was determined via two-way analysis of variance. *P* values less than 0.05 were considered statistically significant, and the significance was indicated on the graphs with asterisks.

## 3. Results

### 3.1. The cytoprotective effect of rutin in HepG2 cells and zebrafish larvae

**Figure 1.**
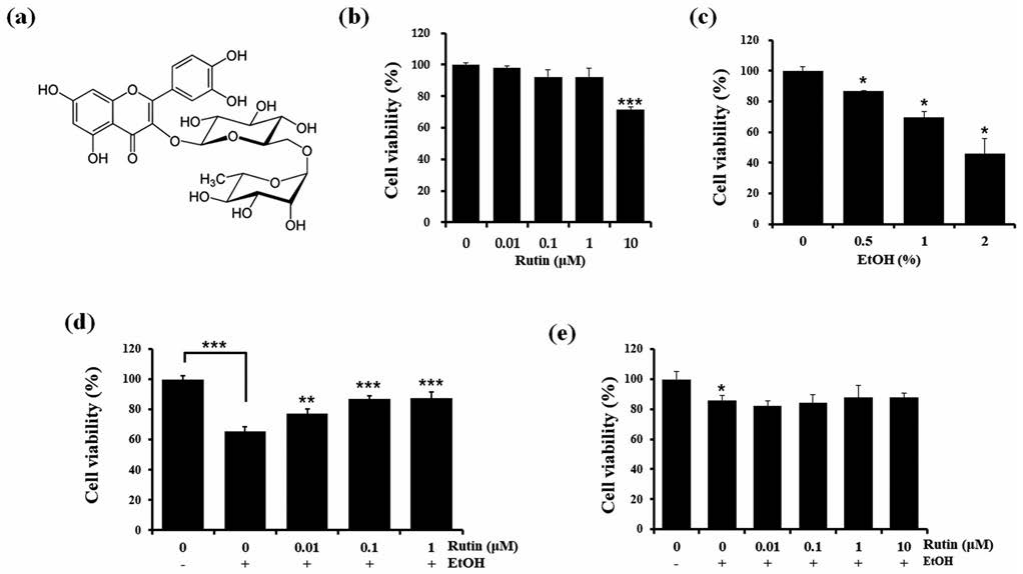
Chemical structure of rutin (a).and cell viability of rutin and ethanol treated HepG2 cells and zebrafish larvae. (b) Viability of HepG2 cells treated with different concentrations of rutin (0–10 μM) for 1 day. (c) Viability of HepG2 cells at different concentration of ethanol (0–2%). (d) Viability of HepG2 cells treated with ethanol and rutin. (e) MTT assay of zebrafish larvae (4 dpf) incubated with different concentrations of rutin (0–1 μM) and 2% ethanol for 24 h. Data are means ± SEM of triple independent experiments. *p < 0.05, **p < 0.01, and ***p < 0.001.

As ethanol consumption is known to damage tissues, we first examined the effects of rutin itself on the viability of HepG2 cells, a human hepatocarcinoma cell line, using the MTT assays (Figure 1(b)). Rutin did not affect HepG2 cell viability at 0.01, 0.1, and 1 μM. However, 10 μM of rutin decreased HepG2 cell viability to about 70% indicating cytotoxicity of rutin at high concentrations. Ethanol decreased cell viability in a dose-dependent manner (Figure 1(c)). Since less than 50% of HepG2 cells survived in the presence of 2% ethanol, we selected 1% ethanol instead for the induction of cytotoxicity. The cytoprotective effect of rutin against ethanol exposure was tested by incubating HepG2 cells with 1% ethanol and 0.01, 0.1, and 1 μM concentrations of rutin for 24 h. Rutin treatment reduced cytotoxicity of HepG2 cells in 1% ethanol (Figure 1(d)). In zebrafish larvae experiments, we selected 2% ethanol due to its lower levels of lethality for zebrafish larvae in order to evaluate the effect of rutin under acute ethanol exposure (Lin et al. 2015). A dose of 1 μM rutin in 2% ethanol did not show serious morphological changes or lethality. Reduction in cytotoxicity was not observed at all rutin concentrations under 2% ethanol (Figure 1(e)) in zebrafish larvae.

### 3.2. Rutin alleviates lipid accumulation induced by ethanol in zebrafish larvae

Zebrafish is an ideal vertebrate model system of human diseases due to strong homology to human genes, as well as ease of monitoring morphological and functional processes. Recent studies have shown that zebrafish process dietary lipids and respond to pharmaceuticals used to treat hypercholesterolemia similar to humans (Schneider et al. 2017). In addition, zebrafish and mammals share similar pathways for the synthesis of lipid signaling molecules, such as prostaglandins and thromboxanes, which can be blocked by general nonsteroidal anti-inflammatory drugs. We therefore utilized the zebrafish larvae system to study ethanol-induced steatosis in acute phase response.

**Figure 2.**
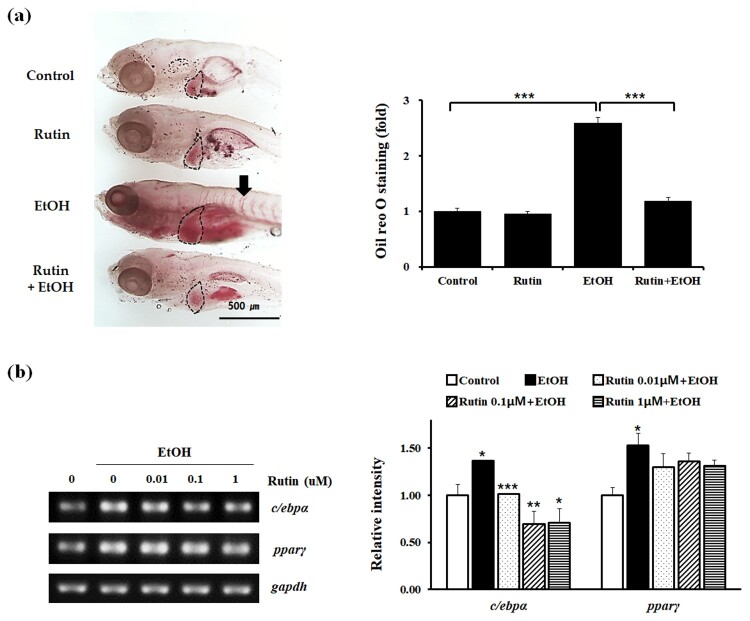
The effect of rutin on hepatic steatosis in zebrafish larvae exposed to ethanol. (a) Representative images of Oil Red O staining. Zebrafish larvae (4 dpf) were incubated with 2% ethanol and/or 1 μM rutin for 24 h and hepatic fatty acid accumulation was measured by Oil Red O density. Marked areas indicate the livers of larvae. Quantitative analysis of relative intensities of Oil Red O staining of larval livers is presented. (b) Zebrafish total RNA was analyzed to determine *c/ebpα* and *ppar*γ expression. Data are shown as means ± SEM of measurements obtained from each of the 20 larvae based on 3 independent experiments. *p < 0.05, **p < 0.01 and ***p < 0.001.

To test the effect of rutin on lipid metabolism in zebrafish, 4 day post fertilization (dpf) larvae were incubated with 1 μM rutin and 2% ethanol for 24 h. The development of hepatic steatosis in larvae was determined by staining with Oil Red O (Figure 2(a)). Control and rutin-treated larvae are rarely stained, whereas the 2% ethanol-treated group stained intensely due to 2.5-fold higher levels of steatosis. The intense Oil Red O staining was strongly detected even in the blood vessels of larvae treated with ethanol. Interestingly, larvae treated with 1 μM rutin and 2% ethanol did not develop steatosis. Rutin treatment significantly reversed steatosis induced by acute ethanol exposure.

The mechanism of action of rutin was investigated further by determining the suppression of lipid metabolism in zebrafish. We analyzed the mRNA levels of transcription factors in adipogenesis via RT-PCR (Figure 2(b)). Ethanol increased the transcription levels of *c/ebpα* and *pparγ* in zebrafish. Rutin significantly suppressed the expression of *c/ebpα* and *pparγ* in ethanol treated larvae. These data confirm that rutin can rescue the phenotype of steatosis associated with ethanol treatment. Taken together, our results suggest that rutin effectively inhibits lipid absorption in alcohol-induced fatty liver of zebrafish.

### 3.3. Rutin rescues mitochondrial network activity via drp1 suppression following ethanol exposure in zebrafish

**Figure 3.**
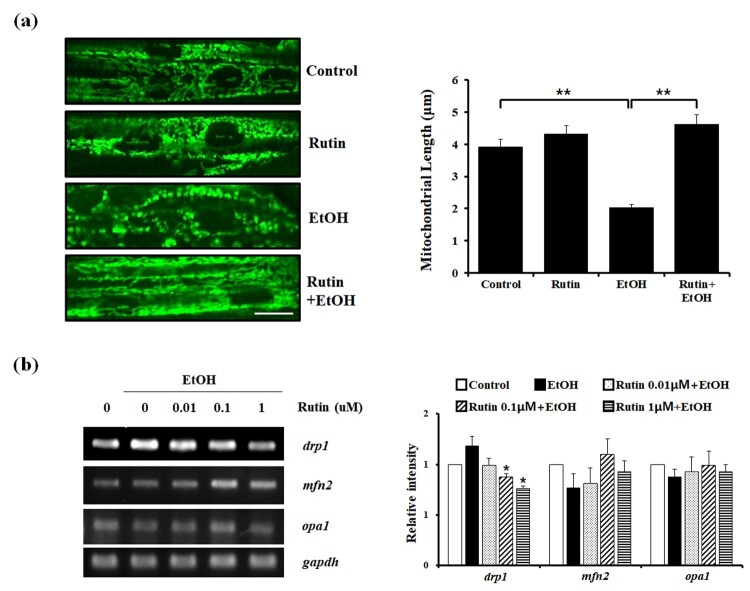
The effect of rutin on mitochondrial dynamics in zebrafish larvae. (a) Representative confocal live images of the mitochondria (green fluorescent) with effect of rutin and ethanol on zebrafish larvae muscle. *TG(mito:EGFP)* zebrafish larvae (4 dpf) were incubated with or without 1 μM rutin under 2% ethanol for 24 h and visualized using a LSM 700 confocal laser microscope (X40). Bar = 10 μm. Quantitative analysis of mitochondrial length in larval muscle cells is presented. Data shown are the means ± SEM of measurements taken from 50 individual cells from three independents experiments. (b) RT-PCR results of mitochondrial fusion and fission related genes affected by rutin following ethanol exposure. (c) Quantitative analysis of relative intensity of *drp1*, *mfn2*, and *opa1* normalized by *gapdh* compared to non-treated cells. Data shown are the means ± SEM of measurements taken from three independent experiments. *p < 0.05 and ***p < 0.001.

Next, we investigated whether rutin has the ability to extend mitochondrial networks from alcohol-induced cytotoxicity *in vivo*. Since the live monitoring of mitochondrial morphology is not easy in the animal systems, *TG(mito:EGFP)* zebrafish were used to trace the changes in mitochondrial dynamics live to study mitochondrial physiology and pathogenesis *in vivo*. We imaged live fish mitochondrial morphology in the liver, but failed to detect EGFP signal under ethanol exposure. Since ethanol exposure is known to cause mitochondrial depolarization in the liver the EGFP fluorescent signal might fade away as well. We visualized live cell imaging of mitochondrial networks in muscle cells of *TG(mito:EGFP)* zebrafish, to evaluate mitochondrial dynamics (Figure 3(a)). Four dpf zebrafish larvae were treated with or without 1 μM rutin and 2% ethanol for 24 h. Live cell imaging of *TG(mito:EGFP)* larvae revealed normal mitochondrial morphology and the average length of mitochondria was ∼ 4 μm in the muscles of control and rutin-treated larvae. The addition of ethanol reduced the length of mitochondria by 50% compared with the control mitochondria. In the presence of 1 μM rutin with ethanol, the length of mitochondria was extended ∼ 2-fold compared with mitochondria of ethanol-treated fish. Rutin restored the mitochondrial dynamics in zebrafish larvae following ethanol exposure.

Our findings demonstrating the effect of rutin under ethanol exposure on mitochondrial morphology raises the possibility of mitochondrial morphology might be mediated by mitochondrial fission-fusion events. Since the expression levels of *drp1* (*dynamin-related protein 1*) and *mfn2* (*mitofusin 2*) controls mitochondrial fission and fusion activities (Zamponi et al. 2018), we investigated whether the restoration of mechanism under this phenotype depends on *drp1* and *mfn2* regulation (Figure 3(b)). The mRNA level of *drp1* was significantly increased upon ethanol treatment and significantly decreased nearly 40% in 1 μM rutin treated cells exposed to ethanol compared to the ethanol group. In contrast to reduced *drp1* expression following rutin treatment of cells exposed to ethanol, the expression of *mfn2* was not significantly changed. The mitochondrial fusion machinery was re-evaluated by analyzing the expression of *opa1*, another major player of mitochondrial fusion. In contrast to changes in *mfn2* expression in response to rutin treatment against ethanol, the levels of *opa1* did not change. These data suggest that rutin treatment suppresses the fission mechanism that is dependent on *drp1* expression, without affecting the mitochondrial fusion.

### 3.4. Rutin inhibits ethanol-induced lipid accumulation in HepG27 cells

**Figure 4.**
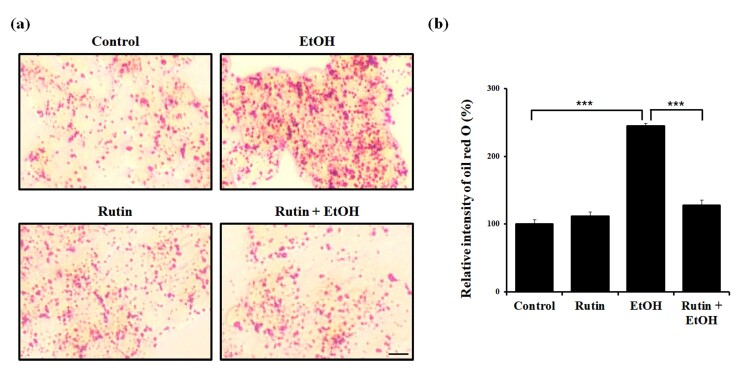
Rutin reduces lipid accumulation on HepG2 cells induced by ethanol. (a) Representative images of Oil Red O. HepG2 cells were incubated with 1% ethanol and/or rutin. Fatty acid accumulation was observed with Oil Red O. Images were acquired with Leica microscope (X40). Bar = 10 μm (b) Quantitative analysis of relative intensities of Oil Red O.

Since ethanol is known to cause steatosis in hepatocytes, we examined the effect of ethanol and rutin in HepG2 cells. Based on Oil Red O staining, the number of lipid droplets (LDs) upon 1% ethanol exposure was dramatically increased in HepG2 cells (Figure 4(a)). Treatment with 1 μM rutin did not affect the number of LDs. However, However, rutin treatment in alcohol exposure reduced the intensity of Oil Red O by 86% compared with ethanol-exposed cells (Figure 4(b)). Taken together, ethanol triggered LDs accumulation and rutin treatment decreased the accumulation of LDs caused by ethanol in HepG2 cells. These findings are compatible with the reduced lipid accumulation in rutin treated zebrafish.

### 3.5. Rutin restores mitochondrial dynamics via suppression of DRP1 under ethanol exposure in HepG2 cells

**Figure 5.**
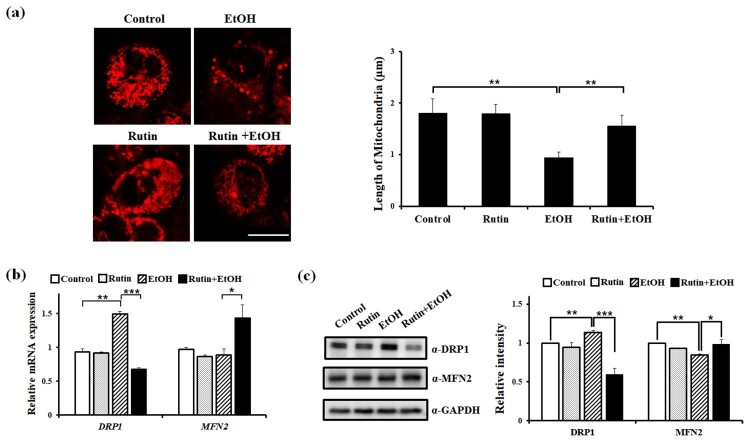
The effect of rutin on mitochondrial dynamics in HepG2 cells (a) Representative confocal live images of mitochondria-targeted DsRed showing the effect of rutin. HepG2 cells transfected with mito-DsRed vector were incubated with 1 μM rutin and/or 1% ethanol for 24 h. Bar = 5 μm. Quantitative analysis of mitochondrial length in HepG2 cells. Data shown are the means ± SEM of measurements obtained from 50 individual cells based on three independent experiments. (b–c) Effects of rutin under ethanol exposure on the expressions of *DRP1* and *MFN2*. (b) Quantitative analysis of relative mRNA intensity of *DRP1* and *MFN2* normalized by GAPDH is presented. (c) Western blot analysis of DRP1 and MFN and quantitative analysis of relative protein intensity of DRP1 and MFN2 normalized by GAPDH are presented Data represents the means ± SEM of measurements obtained from three independent experiments. *p < 0.05, **p < 0.01.

The effect of rutin on mitochondrial dynamics and steatosis under ethanol exposure was evaluated using HepG2 cells expressing mitochondria-targeted DsRed to perform a live imaging of mitochondrial morphology. Cells were incubated with DMSO as a control and 1 μM rutin for 24 h. The length and morphology of mitochondria were analyzed to determine the change in mitochondrial dynamics (Figure 5(a)). The majority of control and rutin treated cells contained mitochondria of normal length (average length about 1.8 μm). No significant difference was observed in rutin only treated cells. Cells with 1% ethanol showed fragmented mitochondria with a 2-fold reduced length compared with that of control cells. Cells with 1 μM rutin and 1% ethanol contained ∼ 1.7 fold longer mitochondria than 1% ethanol treated cells. Ethanol treatment changed the morphology of mitochondria. While HepG2 cells exposed to ethanol showed small and tubular mitochondria, cells treated with rutin under ethanol exposure showed elongated and hyperfused mitochondria compared with ethanol-exposed cells.

Next, we examined the mRNA expression of DRP1 and MFN2 in the mitochondrial dynamics. The level of DRP1 was 46% increased following ethanol incubation, which paralleled the mitochondrial fragmentation detected (Figure 5(b)). Treatment with 1 μM rutin under ethanol exposure for 24 h induced a nearly 60% decrease in DRP1 expression compared to ethanol alone. In contrast to the reduction of DRP1 expression by rutin, the mRNA expression of MFN2 was increased with ethanol and rutin treatment. The protein expression of DRP1 was markedly decreased with rutin and ethanol treatment compared to ethanol-exposed cells (Figure 3(c)). In contrast to DRP1, the expression of MFN2 was slightly increased following following rutin treatment of cells exposed to ethanol. These data imply that ethanol treatment for 24 h altered mitochondrial morphology in HepG2 cells and rutin abrogated mitochondrial abnormalities induced by ethanol due to inhibition of mitochondrial fission via downregulation of DRP1.

## 4. Discussion

AFLD and NAFLD can progress from mild fatty liver to severe steatohepatitis (Shin et al. 2020). Alcohol consumption causes 3 million deaths every year, representing 5.3% of all deaths in worldwide (Sussman and Lucey 2019). Despite the high incidence of AFLD, the pathophysiology of AFLD is poorly understood and mitochondria targeted therapies have not been developed. It is therefore important to develop therapeutic agents and treatments for AFLD. Since the liver tissues in NAFLD patients show mitochondrial dysfunction and oxidative stress (Gong et al. 2019), we identified a therapeutic agent that targets mitochondrial dysfunction in AFLD.

Acute ethanol exposure leads to excessive fission of mitochondria and mitophagy and the imbalance in mitochondrial fission/fusion contributes a significantly to alcohol-related liver diseases. We confirmed that ethanol induced the accumulation of lipid droplets and mitochondrial fragmentation in HepG2 cells. Rutin ameliorates oxidative stress and maintains hepatic and renal functions following exposure to cadmium and ethanol (Abarikwu et al. 2017). Since few pharmacological treatments are available for AFLD, the development of novel therapeutic strategies requires *in vivo* animal models. Here, we utilized the zebrafish model system to investigate the effect of rutin on steatosis following ethanol exposure. Strikingly, rutin reduces the lipid droplet accumulation and restores the mitochondrial networks to normal status. This implies an excellent hepato-protective effect of rutin for the steatosis in zebrafish larvae liver.

To reveal the regulation mechanism of mitochondrial dynamics of rutin, we tested the change in expression of DRP1 for a fission player and MFN2 for a fusion mediator. Defective mitochondrial dynamics causes fat accumulation (Mansouri et al. 2018). Therefore, identifying DRP1 inhibitors may help mitigate mitochondrial dysfunction, cytotoxicity, and maintain cellular homeostasis. For the first time, we suggest that rutin targets mitochondrial dynamics mediated by DRP1 suppression to rescue hepato-cytotoxicity in zebrafish and HepG2 cells. Since DRP1 inactivation in VL-17A cells is known to rescue cell growth retardation from ethanol exposure (Palma et al. 2019), the DRP1 suppression by rutin implies a beneficial effect to reduce liver injury by protecting hepatocytes from alcohol toxicity. Further, recent studies reported that DRP1 inhibition alleviates hepatotoxicity induced by toxic agents like senecionine and cadmium in diet-induced steatosis and liver injury (Zhang et al. 2019). The most important finding of the current study is that rutin inhibited DRP1 to retard fission in zebrafish larvae, as well as HepG2 cells. We present evidence supporting the role of rutin as an inhibitor of DRP1, the master player in mitochondrial fission. We identify DRP1 as a novel target in rutin signaling for treatment of dysregulation of mitochondrial networks. Our results demonstrate that the hepato-protective mechanism of rutin decreases the level of lipid droplets in HepG2 cells, reduces steatosis in the liver of zebrafish larvae, and restores mitochondria fragmentation induced by ethanol. Based on these findings, it is suggested that rutin may serve as a novel therapeutic agent by inhibiting DRP1 to restore mitochondrial dysfunction following ethanol insult.
